# The protective role of the 3-mercaptopyruvate sulfurtransferase (3-MST)-hydrogen sulfide (H_2_S) pathway against experimental osteoarthritis

**DOI:** 10.1186/s13075-020-02147-6

**Published:** 2020-03-17

**Authors:** Sonia Nasi, Driss Ehirchiou, Athanasia Chatzianastasiou, Noriyuki Nagahara, Andreas Papapetropoulos, Jessica Bertrand, Giuseppe Cirino, Alexander So, Nathalie Busso

**Affiliations:** 1 Service of Rheumatology, Department of Musculoskeletal Medicine, Centre Hospitalier Universitaire Vaudois and University of Lausanne, Lausanne, Switzerland; 2 First Department of Critical Care and Pulmonary Services, Faculty of Medicine, National and Kapodistrian University of Athens, Athens, Greece; 3 Laboratory of Pharmacology, Faculty of Pharmacy, University of Athens, Athens, Greece; 4 Isotope Research Center, Nippon Medical School, Tokyo, Japan; 5 Center of Clinical, Experimental Surgery & Translational Research, Biomedical Research Foundation of the Academy of Athens, Athens, Greece; 6 Department of Orthopaedic Surgery, Otto-von-Guericke University, Magdeburg, Germany; 7 Department of Pharmacy, University of Naples Federico II, Naples, Italy

**Keywords:** Calcium-containing crystals, Osteoarthritis, Animal model, Hydrogen sulfide, Chondrocyte calcification

## Abstract

**Background:**

Osteoarthritis (OA) is characterized by the formation and deposition of calcium-containing crystals in joint tissues, but the underlying mechanisms are poorly understood. The gasotransmitter hydrogen sulfide (H_2_S) has been implicated in mineralization but has never been studied in OA. Here, we investigated the role of the H_2_S-producing enzyme 3-mercaptopyruvate sulfurtransferase (3-MST) in cartilage calcification and OA development.

**Methods:**

3-MST expression was analyzed in cartilage from patients with different OA degrees, and in cartilage stimulated with hydroxyapatite (HA) crystals. The modulation of 3-MST expression in vivo was studied in the meniscectomy (MNX) model of murine OA, by comparing sham-operated to MNX knee cartilage. The role of 3-MST was investigated by quantifying joint calcification and cartilage degradation in WT and 3-MST^−/−^ meniscectomized knees. Chondrocyte mineralization in vitro was measured in WT and 3-MST^−/−^ cells. Finally, the effect of oxidative stress on 3-MST expression and chondrocyte mineralization was investigated.

**Results:**

3-MST expression in human cartilage negatively correlated with calcification and OA severity, and diminished upon HA stimulation. In accordance, cartilage from menisectomized OA knees revealed decreased 3-MST if compared to sham-operated healthy knees. Moreover, 3-MST^−/−^ mice showed exacerbated joint calcification and OA severity if compared to WT mice. In vitro*,* genetic or pharmacologic inhibition of 3-MST in chondrocytes resulted in enhanced mineralization and IL-6 secretion. Finally, oxidative stress decreased 3-MST expression and increased chondrocyte mineralization, maybe via induction of pro-mineralizing genes.

**Conclusion:**

3-MST-generated H_2_S protects against joint calcification and experimental OA. Enhancing H_2_S production in chondrocytes may represent a potential disease modifier to treat OA.

## Background

Osteoarthritis (OA) is the most common joint disease affecting millions of people [1]. It is characterized by cartilage degradation, subchondral bone sclerosis and synovitis [2]. In addition, calcium-containing crystals within joint structures are another prominent features of OA, and they participate in its initiation and progression. These crystals were found in 50% up to 100% of synovial fluid [3] and cartilage [4] from OA patients undergoing joint replacement. Two families of calcium-containing crystals were identified in OA: basic calcium phosphate (BCP) (e.g., hydroxyapatite (HA)), and calcium pyrophosphate dihydrate (CPPD) [5]. In vitro, BCP crystals induced catabolic and inflammatory responses [6, 7]. When injected into mice knees, they caused mild synovitis but severe cartilage damage, resembling human OA features [8]. In our recent study, we observed BCP calcific deposits in joints following meniscectomy. Moreover, we found reciprocal crosstalk between BCP and IL-6 production [9], and a positive correlation between these two entities and the severity of cartilage degradation. Thus, we hypothesize that inhibiting crystal formation and deposition in the joint could be of therapeutic value for OA treatment.

Hydrogen sulfide (H_2_S) is an endogenous gasotransmitter in our body, together with nitric oxide (NO) and carbon monoxide (CO) [10]. In mammalian tissues, H_2_S is generated by three different enzymes: cystathionine beta-synthase (CBS), cystathionine gamma-lyase (CSE), and 3-mercaptopyruvate sulfurtransferase (3-MST). These enzymes use cysteine as a substrate to produce H_2_S [11, 12], and their expression is tissue-specific. H_2_S showed biological effects [13] that can be of relevance in OA, such as reduced pro-inflammatory responses [14], reduced mineralization [15–18], improved anabolic/catabolic balance [19, 20], and decreased oxidative stress (reactive oxygen species (ROS) production) [21, 22]. H_2_S signaling occurs in part through post-translational modification (namely, S-sulfhydration) of specific cysteine residues in target proteins with the potential to alter their function [23]. In addition, H_2_S oxidation leads to sulfite (SO_3_^2−^), thiosulfate (S_2_O_3_^2−^) [24], and sulfate (SO_4_^2−^) generation in the mitochondria [12], which could themselves mediate H_2_S effects.

Current therapeutic approaches for OA are either symptomatic or surgical. Therefore, there is a medical need for interventions that target the pathological processes of OA. We hypothesized that H_2_S can prevent calcium-containing crystals deposition in the joint, and subsequently OA progression. In particular, we demonstrated that activation of the 3-MST/H_2_S axis improved outcomes in both human and experimental OA.

## Methods

### Mice and experimental osteoarthritis

3-MST KO (*n* = 8) [25] and WT female mice (*n* = 8), 8-weeks old, on a C57BL/6 background, were subjected to medial meniscectomy (MNX) of the right knee, while the contralateral knee was sham-operated as control [26]. Two months after, mice were sacrificed, blood collected, and serum obtained by 15 min centrifugation at 15000×*g*, and knees fixed in 10% formalin.

### MicroCT-scan

MicroCT-scans analysis was performed using a SkyScan 1076® X-ray μCT scanning system (SkyScan, Belgium) and the following parameters: 18 μm resolution, 60 kV, 167 μA, 0.4° rotation step over 360°, 0.5 mm Aluminum filter, 1180 ms exposure time. Ex vivo samples acquisition was made using formol fixed knees. Images were reconstructed using NRecon Version 1.6.6.0 (Skyscan, Belgium) considering the following parameters: gray-values = 0.0000–0.105867, ring artifact reduction = 3, beam hardening correction = 40% [27]. Newly formed calcific deposits at the site of the removed medial meniscus were considered as Volumes-Of-Interest (VOI) for the quantitative analysis of new formation volume (mm^3^) and new formation crystal content (μg) by CTAnalyzer V.1.10.

### Mouse knee histology

Knees were decalcified in EDTA for 20 days and embedded in paraffin. Sagittal sections (5 μm thick, 3 sections/mouse, spaced 70 μM apart) of the medial compartment were stained with Safranin-O and counterstained with fast green/iron hematoxylin. Blinded OARSI score (0–24 score) [28] for cartilage damage and Safranin-O loss was assessed by two independent observers.

### Thiosulfate measurement

Serum was delipidized with dichloromethane and centrifuged. The supernatant was derivatized with monobromobimane, acetonitrile, and HEPES/EDTA buffer (pH 8) for 30 min in the dark. Methanosulfonic acid was added to stop the reaction and proteins removed by centrifugation. Thiosulfate was determined by HPLC [29, 30]: Waters-2695 module, fluorescence detector (excitation wavelength of 380 nm, the emission wavelength of 480 nm) and a reverse-phase column. The eluants were PIPES (10 mM, pH 6.6) and methanol (gradient). Concentrations were calculated by integrating the area under the curve.

### Human cartilage explants

Human cartilage (tibia and femur) from 15 OA patients undergoing knee replacement (Kellgren-Lawrence K/L score 1 to 4, age 64.69 years ± 10.58) was obtained from the Otto-von-Guericke University (Magdeburg-D). Patients were grouped into low (K/L 1–2 and OARSI 2–3, age 70.25 ± 5.37 years), medium (K/L 3 and OARSI 3–4, age 65.25 ± 14.88 years), and high (K/L 4 and OARSI 5, age 59.8 ± 9.33 years) OA grade. Full-thickness cartilage explants were fixed in 4%PFA and embedded in paraffin. Five-micrometer-thick sagittal sections were cut for further immunohistochemical and calcification analysis.

For HA crystal stimulation experiment, cartilage (tibia and femur) from 4 OA patients (mean age 72 ± 10 years) undergoing knee replacement (K/L score = 4) was obtained from the Orthopedic Department (CHUV, Lausanne-CH). Six-millimeter-diameter disks (3 disks/patient) were dissected from macroscopically intact cartilage using a dermal punch. In order to match for location across treatment groups, each disk was divided into two equal parts, and each half was stimulated or not with 500 μg/ml HA crystals for 24 h in DMEM+ 1%P/S + 50 μg/ml L-ascorbic acid 2-phosphate. Cartilage was fixed in 4%PFA for immunohistochemical analysis.

### Human cartilage histology and quantification of calcification

For each patient, three sections of full-thickness cartilage were stained with Von Kossa/Safranin-Orange staining (Sigma). Pictures were taken using a Zeiss Axiovert microscope and Zen software at × 2.5 magnification, in order to have the whole cartilage section depicted on the picture. Images were then converted into a grayscale. The total cartilage area (100%) was marked in the image using ImageJ (NIH Image). The percentage (%) of calcified cartilage over the total cartilage area was identified using a threshold for black and white. The mean value of the three sections/patient was calculated. Four to 5 patients were analyzed for each K/L-OARSI group.

### Immunohistochemical analysis

3-MST expression was evaluated using an anti-3-MST rabbit polyclonal antibody (Novusbio NBP1-82617) on paraffin sections. The antibody was demonstrated to be specific, as a negative staining was obtained both when 3-MST IHC was performed without the primary antibody (data not shown) and when 3-MST IHC was performed on knee sections from 3-MST KO mice (Fig. 2a, Sham 3-MST KO).

Analysis of 3-MST expression in sham-operated versus meniscectomized murine knees was made by evaluation of positivity in histological sections. In humans, for each patient, three sections of full-thickness cartilage were stained with the 3-MST Ab. Pictures of three fields per section were taken using a Zeiss Axiovert microscope and Zen software at × 10 magnification. The total number of cells and the number of 3-MST-positive cells were counted in each field, and the percentage (%) of 3-MST-positive cells was calculated. The mean value of the three fields was calculated for each section and the mean value of the three sections/patient was calculated. Four to 5 patients were analyzed for each K/L-OARSI group.

### Hydroxyapatite crystals and secondary calciprotein particles

Hydroxyapatite crystals (HA) crystals were synthesized, characterized [31], and sonicated for 5 min in sterile PBS prior to experiment. Secondary calciprotein particles (CPP) were synthesized as previously described [15]. Briefly, 10% FBS, 3.5 mM phosphate (2.14 mM Na_2_HPO_4_, 1.36 mM NaH_2_PO_4,_ Sigma), 1 mM calcium (CaCl_2_, Sigma), 1%P/S, and 1%L-Glutamine where added to DMEM. This medium was stored at 37 °C for 7 days to generate secondary CPP and then centrifuged at 25000×*g* for 2 h at 4 °C. Calcium content was measured in the resuspended pellet by the QuantiChrom™ Calcium Kit.

### Murine articular chondrocytes isolation

Primary knee immature chondrocytes were isolated from 5 to 7 days old mice [9] and amplified for 7 days in DMEM+ 1%P/S + 10%FBS to reach chondrocytic differentiation [32]. For calcification studies, chondrocytes were cultivated for 24 h in DMEM+ 1%P/S + 10%FBS, supplemented with secondary calciprotein particles (CPP-50 μg/ml calcium) to induce calcification and were concomitantly treated with 0.4% DMSO, 500 μM H_2_O_2_ (Sigma-Aldrich, dissolved in culture medium), 1 mM N-acetylcysteine NAC (Sigma-Aldrich, dissolved in DMSO), or a combination of those. For qRT-PCR studies, separate plates were used and chondrocytes were cultivated for 4 h in DMEM+ 1%P/S only, supplemented with 0.4% DMSO or 50 μM of the 3-MST inhibitor (compound 3 [33], dissolved in DMSO, kindly provided by Prof. Kenjiro Hanaoka, University of Tokyo). For alkaline phosphatase activity, separate plates were used and chondrocytes were cultivated for 6 h in DMEM+ 1%P/S only, supplemented with 0.4% DMSO, 50 μM of the 3-MST inhibitor, 500 μM H_2_O_2,_ or a combination of those.

### Crystal detection in articular chondrocyte cultures

For Alizarin Red staining, cells were fixed in 10% formol for 30 min and calcium-containing crystals stained by applying 2% Alizarin red solution (pH 5.3) for 1 h [34]. After washings with tap water, pictures were taken. For calcium content quantification, separate plates were used. Cell monolayers were decalcified with 0.6 M HCl for 24 h. The following day, calcium content was quantified by the QuantiChrom™ Calcium Kit (BioAssay Systems) by reding absorbance at 612 nm using the Spectramax M5e reader (Molecular Devices).

### IL-6 quantification in articular chondrocyte cultures

Cell supernatants from the cells used for the measurement of calcium content were assayed using murine IL-6 ELISA kit (eBioscience) and by reading absorbance at 450 nm and 570 nm using the Spectramax M5e reader.

### Alkaline phosphatase (Alp) activity in articular chondrocyte cultures

Supernatant was removed, chondrocytes lysed in 0.01% SDS (dissolved in water), and alkaline phosphatase (Alp) activity was measured in cell lysate using a p-Nitrophenyl Phosphate assay (Alpl Assay Kit, Abcam, ab83369) and by reading absorbance at 405 nm.

### H_2_S detection in articular chondrocyte cultures

Primary murine chondrocytes (10^6^cells/condition) were treated for 6 h with 50 μM 3-MST inhibitor or 0.4% vehicle (DMSO). They were then resuspended in fluorescence-activated cell sorting (FACS) buffer (5%FCS, 5 mM EDTA in PBS) and the H_2_S fluorescent probe P3 added (10 μM, [35]). FACS analysis was performed right after with a UV laser (LSRII SORP cytometer, BD Biosciences) and data processed by FACS Diva (BD Biosciences) and FlowJoX (Tree Star).

### LDH measurement in articular chondrocyte cultures

Measurement of the leakage of components from the cytoplasm into the surrounding culture medium has been widely accepted as a valid method to estimate the number of non-viable cells. Lactate dehydrogenase (LDH) in the supernatant was measured using the fluorimetric method CytoTox-ONE™ Homogeneous Membrane Integrity Assay (Promega), by recording fluorescence at an excitation wavelength of 560 nm and an emission wavelength of 590 nm. Culture medium from wells without cells was used as a negative control (0% cytotoxicity), while medium from wells with lysed cells (1% Triton X-100) was used as a positive control (100% cytotoxicity). The percent cytotoxicity of each experimental wells was then calculated as follows: Percent cytotoxicity = [(Experimental value)-(Culture medium value)]/[(Positive control value)-(Culture medium value)]× 100.

### ATDC5 chondrogenic cell line

For qRT-PCR analysis, ATDC5 cells (from ATCC cell line) were cultured in DMEM/F12 (1%P/S only) and treated for 4 h with different combinations of 0.4% DMSO (Sigma-Aldrich), 50 μl/ml CPP, 50 μM 3-MST inhibitor, and 500 μM H_2_O_2_. For reactive oxygen species (ROS) measurement, separate plates were used and ATDC5 were cultured in DMEM/F12 without phenol red and FBS and cells were treated with the same conditions described above, or with 10 ng/ml mouse recombinant IL-6 (Gibco, dissolved in DMSO) where indicated.

### ROS level measurement in ATDC5 cells

Mitochondrial ROS level was measured with Red Mitochondrial Superoxide Indicator (MitoSOX, Life Technologies). Briefly, ATDC5 in half area 96-wells clear bottom black plate were stimulated or not for 1 h with 50 μl/ml CPP and treated or not with vehicle 0.4% DMSO, 50 μM 3-MST inhibitor, 50 μM H_2_O_2_, 1 mM NAC, or 10 ng/ml IL-6 in DMEM/F12 without phenol red. After stimulation, cells were loaded 30 min with 5 μM MitoSOX, and fluorescence intensity measured (excitation wavelength of 510 nm, emission wavelength of 580 nm) using the Spectrax M5e reader.

Wells with cells-DMEM/F12 only, as well as wells with MitoSOX-DMEM/F12 only, were also included in order to measure cells and MitoSOX autofluorescence, respectively. These background values were then subtracted to the experimental wells values, and the obtained results were plotted as MitoSOX signal (A.U).

### Real-time PCR analysis in articular chondrocyte cultures and ATDC5 cells

Cells were lysed in TRIzol reagent (Thermo Scientific) in a ratio of 500 μl TRIzol every 10^6^ cells. RNA was extracted (RNA Clean and Concentrator5, Zymoresearch), reverse transcribed (Superscript II, Invitrogen), and quantitative Real Time-PCR (qRT-PCR) with gene-specific primers (Table 1) using the LightCycler480®system (Roche Applied Science) was performed. Each reaction mix was composed by 3.75 μl LightCycler 480 SYBR Green I Master (Roche) + 0.75 μl of 5 μM primer pair specific for each gene+ 0.5 μl of LightCycler Water (Roche) + 2.5 μl of 20 ng/μl cDNA. Wells with RNase/DNase-free water instead of cDNA were included for each amplified gene as a negative control.

**Table 1:** Murine gene specific primers for qRT-PCR

Gene	Forward primer (5′ ➔ 3′)	Reverse primer (5′➔ 3′)
*mAnk*	TGT CAA CCT CTT CGT GTC CC	GAC AAA ACA GAG CGT CAG CG
*mAlpl*	TTG TGC CAG AGA AAG AGA GAG	GTT TCA GGG CAT TTT TCA AGG T
*mAnx5*	CCT CAC GAC TCT ACG ATG CC	AGC CTG GAA CAA TGC CTG AG
*mPit-1*	CTC TCC GCT GCT TTC TGG TA	AGA GGT TGA TTC CGA TTG TGC
*mPit-2*	AAA CGC TAA TGG CTG GGG AA	AAC CAG GAG GCG ACA ATC TT
*m3-Mst*	CTG GGA AAC GGG GAG CG	GCT CGG AAA AGT TGC GGG
*mTbp*	CTT GAA ATC ATC CCT GCG AG	CGC TTT CAT TAA ATT CTT GAT GGT C
*mGapdh*	CTC ATG ACC ACA GTC CAT GC	CAC ATT GGG GGT AGG AAC AC

Data was normalized against *Tbp* and *Gapdh* references genes, with fold induction of transcripts calculated against control cells.

### Statistical analysis

For human ex vivo experiments, values represent means ± SD and 4 to 5 patients per group were analyzed. For in vitro experiments, values represent means ± SD of triplicates. For each readout, three independent experiments were performed. For in vivo experiments, eight mice per group were used.

Data was analyzed with GraphPad Prism software. The variation between data sets was evaluated using Student’s *t* test or one-way or two-way ANOVA test, where appropriate. Bonferroni correction was used as a post hoc analysis in case of multiple comparisons. Correlation between parameters was evaluated using the correlation test and expressed by the Pearson correlation coefficient (− 1 < *r* < 1). Differences were considered statistically significant at *p < 0.05, **p < 0.01, ***p < 0.001, and ****p < 0.0001.

## Results

### 3-MST expression in human cartilage negatively correlates with OA severity and chondrocyte calcification, and it is downregulated by HA crystals

**Fig. 1:**
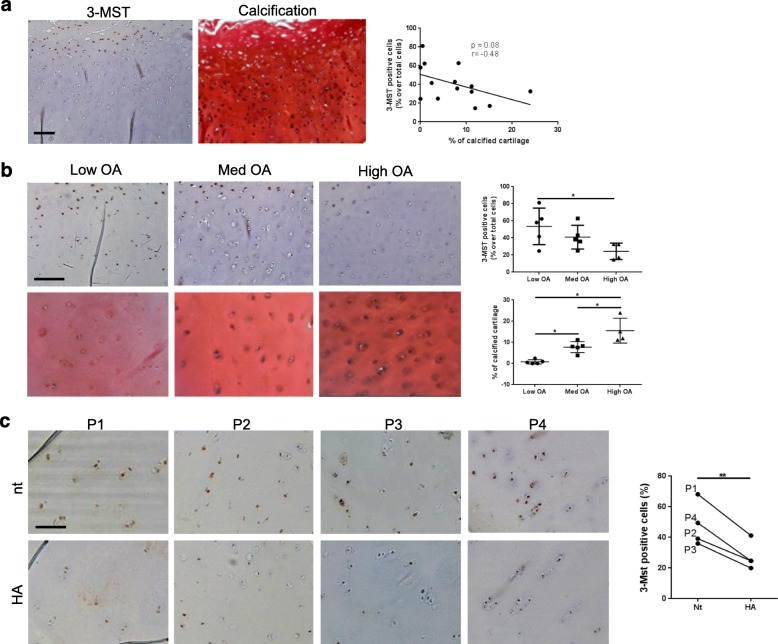
Cartilage 3-MST expression is negatively correlated with OA severity and chondrocyte calcification and downmodulated by HA crystals. **a** 3-MST immunohistochemical staining in cartilage explants from end-stage osteoarthritis patients and consecutive sections stained with von Kossa/Safranin-O staining for calcium-containing crystals. For each staining, one representative picture from one out of 14 patients is shown. Scale bars 200 μm. The graph shows the correlation between the % of 3-MST-positive cells and the % of calcified cartilage in the different patients. *n* = 14 patients. **b** Immunohistochemical staining of 3-MST in cartilage from individuals with low, medium, and high stage osteoarthritis and von Kossa/Safranin-O staining for calcium-containing crystals in consecutive sections. Pictures from one representative patient out of 5 patients per group are shown. Scale bars 200 μm. The graphs show the % of 3-MST-positive cells and the % of calcified cartilage. Three fields were counted per patient and the mean plotted in the graph. *n* = 14 patients. **c** 3-MST immunohistochemistry of human cartilage explants stimulated 24 h with HA crystals (HA 500 μg/ml) or not (Nt). Scale bars 200 μm. The graph shows the % of 3-MST-positive cells in Nt vs HA-treated explants in each patient. Lines connect the Nt condition and the HA condition for each patient. *n* = 4 patients

Immunohistochemistry on human OA cartilage (Fig. 1a) revealed high 3-MST expression by chondrocytes in the superficial area of cartilage and low expression in intermediate-deep layers. In contrast, chondrocyte calcification was present in deep cartilage and negative in the superficial zone. Thus, we found a trend (*p* = 0.08) towards an inverse correlation (*r* = − 0.48) between the two parameters (graph Fig. 1a). When specimens were divided into low, medium, or high OA, 3-MST expression was decreased by 20–30% in medium and high OA (Fig. 1b), while chondrocyte calcification was increased as assessed by von Kossa staining (Fig. 1b). We next stimulated cartilage explants with HA crystals for 24 h (Fig. 1c). 3-MST expression was significantly inhibited by HA crystals in four patients, although at different degrees (Fig. 1c). Altogether, these results indicate that chondrocyte calcification increases during OA progression and negatively impacts on 3-MST expression proportionally to disease severity.

### 3-MST regulates joint calcification and cartilage damage in experimental OA

**Fig. 2:**
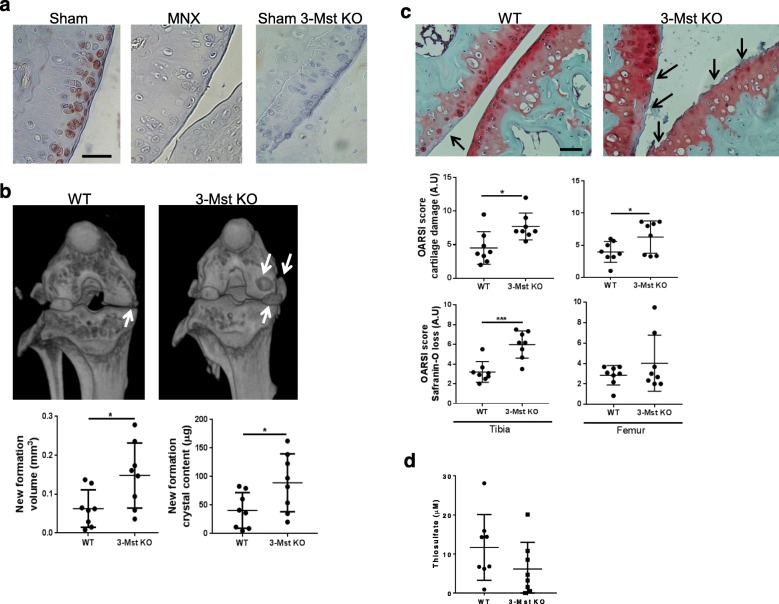
3-MST deficiency exacerbates joint calcification and cartilage damage in experimental OA. **a** Representative immunohistochemical analysis of 3-MST expression in the knee section from sham-operated and MNX WT mice. A knee section from a sham-operated 3-Mst KO mouse was used to prove the specificity of the 3-MST antibody. Scale bars 150 μm. **b** Representative micro-CT scan images of WT and 3-MST KO MNX murine knee joints two months after surgery. White arrows show calcified periarticular deposits in MNX WT knees and their exacerbation in 3-MST KO mice. Graphs show CTAnalyzer quantitative analysis of new formation volume (mm^3^) and new formation crystal content (μg) in WT and 3-MST KO MNX knees. **c** Representative histologies of WT and 3-MST KO MNX knees, stained with Safranin-O. Black arrows show increased cartilage degradation in 3-MST KO mice. Scale bars 150 μm. Graphs show femoral scoring of cartilage damage and Safranin-O loss, accordingly to OARSI method. **d** Thiosulfate measurement in the serum of WT and 3-MST KO mice. Mice number WT *n* = 8, 3-MST KO *n* = 8

To investigate the role of 3-MST in the pathogenesis of osteoarthritis, we subjected 3-MST deficient mice (3-MST KO) to meniscectomy. As in human OA samples, we observed that 3-MST expression was higher in sham-operated healthy knees than in osteoarthritic MNX knees (Fig. 2a). Two months post-surgery, CT-scans evidenced increased calcification in 3-MST KO knees if compared to WT knees (Fig. 2b, white arrows). Quantitative analysis of calcifications revealed that both their volume and their overall crystal content were significantly higher in 3-MST KO mice (graphs Fig. 2b). In parallel, cartilage damage (fissures and fibrillations, black arrows) as well as proteoglycan loss (reduced Safranin-O staining) were exacerbated in 3-MST KO joints compared to WT joints, as mirrored by both histological analysis and OARSI scores (Fig. 2c). Finally, serum thiosulfate level was higher in WT mice than in 3-MST KO mice (Fig. 2d).

### 3-MST regulates chondrocyte mineralization and IL-6 secretion in vitro

**Fig. 3:**
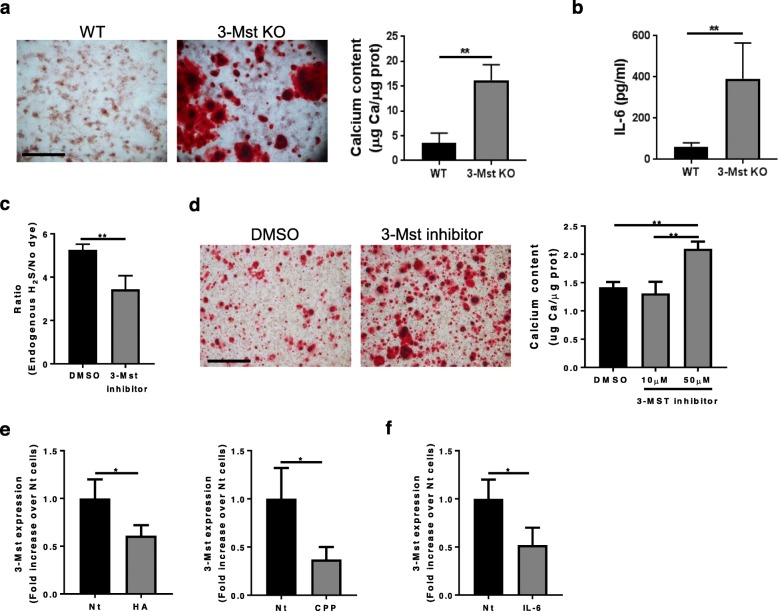
Endogenously H_2_S produced by 3-MST regulates chondrocytes calcification and IL-6 secretion and vice versa. **a** Alizarin red staining of WT and 3-MST KO chondrocytes cultured with CPP for 24 h. Pictures represent triplicates from one experiment of three independent experiments. Graph represents calcium content in the cell monolayer, expressed in μg Ca/μg protein. *n* = 3. **b** IL-6 secretion in cell supernatant of WT and 3-MST KO chondrocytes from point (**a**). *n* = 3. **c** FACS analysis of endogenous H_2_S production by WT chondrocytes treated for 6 h with vehicle (DMSO) or 50 μM 3-MST inhibitor and incubated with P3 probe. *n* = 3. **d** Alizarin red staining of WT chondrocytes cultured in CPP for 24 h in the presence or absence of 50 μM 3-MST inhibitor. Pictures represent triplicates from one experiment of three independent experiments. Graph represents calcium content in the cell monolayer, expressed in μg Ca/μg protein. *n* = 3. **e** qRT-PCR for *3-Mst* gene expression in WT chondrocytes stimulated or not with 500 μg/ml HA crystals or CPP for 4 h, or with (**f**) 10 ng/ml IL-6. *n* = 3

After 24 h in the presence of CPP, 3-MST KO chondrocytes had exacerbated mineralization as demonstrated by Alizarin red staining (Fig. 3a) and by quantification of calcium content in the cell monolayer (graph Fig. 3a). In parallel, we found that 3-MST KO cells secreted higher levels of basal IL-6 than WT cells (Fig. 3b). This would support our previous finding that IL-6 sustains mineralization in chondrocytes [9]. As the second approach to lower 3-MST activity, we treated WT chondrocytes with a pharmacological 3-MST inhibitor. Firstly, we confirmed by FACS that the 3-MST inhibitor significantly decreased H_2_S production by chondrocytes (Fig. 3c). In accordance with the findings in 3-MST KO chondrocytes (Fig. 3a), treatment with the 3-MST inhibitor triggered chondrocyte mineralization (Fig. 3d). Conversely, stimulation of chondrocytes with crystals (HA or CPP) caused 3-MST downregulation (Fig. 3e). Accordingly, the addition of IL-6 also decreased 3-MST expression by 2-fold (Fig. 3f). Altogether these results suggest that two of the main OA triggers (calcium-containing crystals and IL-6) negatively affect the endogenous generation of H_2_S by 3-MST and vice-versa.

### Oxidative stress regulates 3-MST expression and mineralization in chondrocytes

**Fig. 4:**
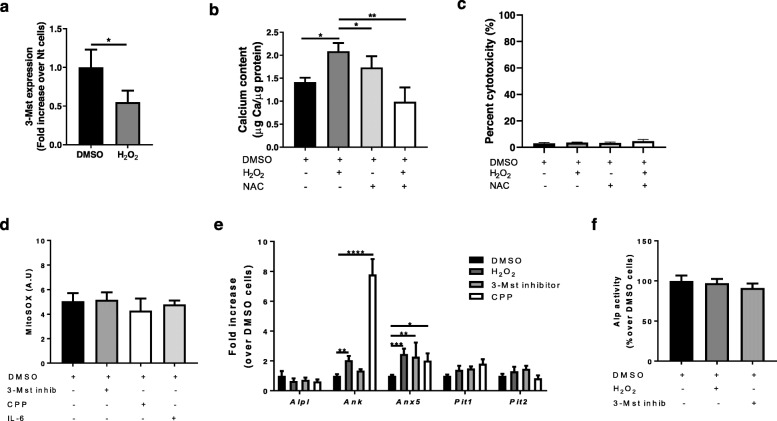
Oxidative stress regulates 3-MST expression, mineralization, and inflammation in chondrocytes. **a** qRT-PCR for *3-Mst* gene expression in WT chondrocytes stimulated or not with 500 μM H_2_O_2_ for 4 h. *n* = 3. **b** Calcium content in chondrocytes monolayer incubated for 24 h with CPP and treated with vehicle (DMSO) or 500 μM H_2_O_2_ or with 1 mM NAC or with a combination of them. Calcium content is expressed in μg Calcium/μg protein. **c** LDH release in cell supernatant of chondrocytes from point (**b**). *n* = 3. **d** Mitochondrial ROS production (MitoSOX) in chondrocytes treated with vehicle (DMSO), or 50 μM 3-MST inhibitor or CPP or 10 ng/ml IL-6 for 1 h. *n* = 3. **e** qRT-PCR of the indicated genes in chondrocytes stimulated with vehicle (DMSO), or 500 μM H_2_O_2_, or 50 μM 3-MST inhibitor or CPP for 4 h. *n* = 3. **f** Alp activity in chondrocytes lysates treated with vehicle, or 500 μM H_2_O_2_, or 50 μM 3-MST inhibitor for 6 h. *n* = 3

Oxidative stress has been implicated in the progression of OA via different mechanisms [36], but never via a direct role in chondrocyte calcification. We found that H_2_O_2_ stimulation led to significantly decreased 3-MST expression (Fig. 4a) in chondrocyte, but concomitantly increased calcification (Fig. 4b), while the ROS scavenger NAC reverted the latter effect. Cell viability was not affected in any conditions (Fig. 4c). Inversely, neither promoters of calcification (CPP and IL-6) nor H_2_S inhibition (3-MST inhibitor) altered mitochondrial ROS production by chondrocytes (Fig. 4d). Taken together, these results support a deleterious role of ROS upstream to chondrocyte calcification and inflammation, likely mediated by inhibition of 3-MST-generated H_2_S.

Finally, we assessed the expression of genes involved in the calcification process in chondrocyte cultured in presence of CPP, or H_2_O_2_, or 3-MST inhibitor. H_2_O_2_ significantly increased *Ank* and *Anx5* expression, as the pro-calcifying stimulus CPP and the 3-MST inhibitor did (Fig. 4e). This strengthens the hypothesis that H_2_O_2_ may induce chondrocyte calcification via inhibition of endogenous H_2_S production. *Alpl* expression (Fig. 4e) and activity (Fig. 4f), and *Pit1* and *Pit2* expression were not modulated in all conditions.

## Discussion

A large body of evidence supports the idea that calcium-containing crystals are active players in the initiation and progression of OA [5, 9, 37, 38]. However, the mechanisms of cartilage calcification are largely unknown and to date, there is no treatment that can prevent crystal deposition or dissolve already formed calcifications.

Here, we have demonstrated that the 3-MST/H_2_S pathway is involved in cartilage calcification and OA progression. Decreasing the endogenous level of H_2_S in chondrocytes, either genetically by 3-MST deficiency or pharmacologically by an 3-MST inhibitor, led to exacerbated mineralization in vitro (Fig. 3a, d). A proof of concept of the protective role of 3-MST-generated H_2_S was given by the in vivo MNX model, where 3-MST-deficient mice were affected by joint calcification and cartilage degradation (Fig. 2b, c) more severely than WT mice. Another evidence was that in knee cartilage from both MNX mice (Fig. 2a) and OA patients (Fig. 1b) we found an inverse correlation between 3-MST expression, and the extent of calcification as well as OA severity.

3-MST deficiency in humans is responsible for a rare inheritable disorder called mercaptolactate-cysteine disulfiduria (MCDU) [39]. MCDU patients are not only mainly affected by mental retardation [25], but can also exhibit skeletal abnormalities (high forehead, arachnodactyly, genum valgum, and joint hyperflexibility (Orphanet:1035)). Other studies support a protective role of H_2_S in pathological mineralization. H_2_S decreased vascular calcification [15, 17, 18, 40], while inhibition of CSE activity caused the opposite effect [18]. The H_2_S-donor sodium thiosulfate (STS) inhibited knee joint calcification during experimental OA [37]. On the other hand, some studies demonstrated the pro-mineralizing effect of H_2_S. H_2_S induced physiological mineralization of human periodontal [41] and mesenchymal [42] stem cells. CBS deficiency was associated with human [43] and murine [44] osteoporosis. Furthermore, the H_2_S-donor GYY4137 stimulated bone formation in vivo [45, 46].

While all these studies investigated the CBS/H_2_S or the CSE/H_2_S pathway in mineralization, to our knowledge, we are the first to highlight the importance of the 3-MST/H_2_S pathway in this context. We will discuss here below the mechanisms by which lack of 3-MST-generated H_2_S could facilitate chondrocyte calcification and OA progression and the time-course of the events.

The very first mechanism involved seems to be reduced 3-MST expression/activity by increased oxidative stress. We indeed showed here that hydrogen peroxide (H_2_O_2_), a major reactive oxygen species (ROS), was able to decrease *3-Mst* expression (Fig. 4a). The effect of ORS on 3-MST can also occur at the post-transcriptional level, as it was shown previously that H_2_O_2_ inhibited the activity of mouse recombinant 3-MST and further H_2_S generation [47]. Subsequently to 3-MST inhibition, we showed that H_2_O_2_ exacerbated chondrocyte mineralization (Fig. 4b) while the ROS scavenger NAC reverted this effect. Other studies exist in the literature that supports an important role or ROS in triggering chondrocyte calcification [48] and metalloproteases production [49, 50], ultimately leading to OA progression. The fact that preventing oxidative stress is beneficial in reducing chondrocyte calcification, was also highlighted in a previous study from our group, in which we demonstrated that the H_2_S metabolite thiosulfate was able to decrease ROS production and calcification in chondrocytes [37]. We therefore hypothesize that increased oxidative stress inhibits 3-MST/H_2_S ultimately leading to increased chondrocyte calcification. Importantly, while ROS suppressed 3-MST/H_2_S pathway and induced calcification, we could not found the opposite, that is 3-MST inhibition or calcification trigger (CPP) did not increase mitochondrial ROS production. This could be because the 3-MST function in mitochondria is compensated by another enzyme called rhodanese [51]. Further investigations are needed to determine if decreased 3-MST/H_2_S impact on total ROS production in chondrocytes.

We next investigated in more details the possible underlying mechanisms by which 3-MST inhibition could exacerbate calcification in chondrocytes, and found that both 3-MST inhibitors (H_2_O_2_ and the 3-MST inhibitor itself) caused upregulation of calcification genes such as *Ank* and *Anx5* (Fig. 4e)*.* This is in line with our previous data of *Anx5* downregulation by the H_2_S metabolite thiosulfate [37]. The expression or the activity of other calcification enzymes and channels (*Alpl*, *Pit1*, *Pit2*) were not impacted by H_2_O_2_ or the 3-MST inhibitor (Fig. 4e, f).

**Fig. 5:**
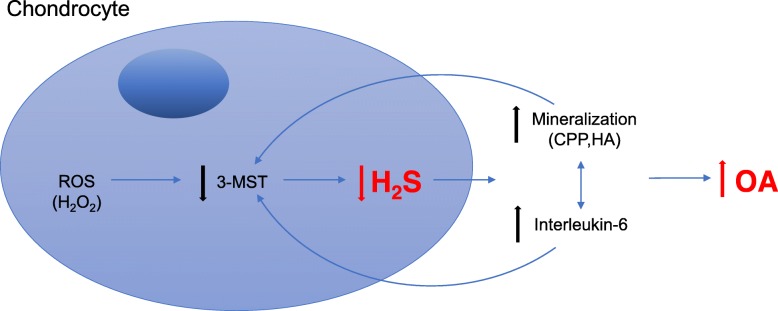
Proposed mechanism for 3-MST involvement in osteoarthritic joints. Firstly, reactive oxygen species such as H_2_O_2_ decrease 3-MST expression (Fig. 4a). 3-MST inhibition leads to decreased endogenous H_2_S production (Fig. 3c) which favors increased chondrocyte calcification (Fig. 3a and d), and interleukin-6 secretion (Fig. 3b). An amplification loop exists between mineralization and interleukin-6, which triggers OA progression [9]. Finally, mineralization and interleukin-6 can also cause downregulation of 3-MST, leading to sustained deleterious signal towards disease progression

An additional trigger of chondrocyte calcification is known to be inflammation. In particular, in our study from 2015 [9], we demonstrated that a vicious cycle exists between chondrocyte calcification and the pro-inflammatory cytokine IL-6. In the current study, we demonstrated that 3-MST inhibition (3-MST deficient chondrocytes), led not only to increased calcification but also to increased IL-6 secretion by chondrocytes (Fig. 3b). Conversely, we found that both pro-calcifying factors (HA, CPP) and pro-inflammatory factors (IL-6) led to the downregulation of *3-Mst* expression (Fig. 3e, f), thus reducing H_2_S. This loop is shown in Fig. 5.

A thing that remains to be clarified is whether the effects caused by inhibition of the 3-MST (exacerbated calcification and inflammation) are due to decreased levels of H_2_S or one of its metabolites such as thiosulfate. In 3-MST KO mice, which have decreased serum levels of H_2_S [25], we also found decreased serum levels of thiosulfate (Fig. 2b). We previously demonstrated that thiosulfate is protective against joint calcification and cartilage degradation in experimental OA, likely due to its anti-inflammatory, antioxidant, and anti-catabolic properties [37].

Finally, further data are needed to determine whether the other H_2_S producing enzymes have a role in calcification in OA. Although we have already excluded a major role for CBS (because not expressed in cartilage, and because we did not observe any OA phenotype in CBS KO mice, data not shown), it is likely that CSE, which is expressed by chondrocytes [52], may as well have a role in joint calcification.

## Conclusions

We have established a key role for the 3-MST/H_2_S axis in the regulation of pathological chondrocyte calcification in OA. Oxidative stress is an upstream event leading to reduced 3-MST/H_2_S levels. Impaired 3-MST/H_2_S levels increase chondrocyte calcification and IL-6 secretion. Moreover, calcium-containing crystals and IL-6 can in turn inhibit 3-MST-mediated H_2_S production, resulting in even greater mineralization and OA progression (Fig. 5). Whether these phenotypes are due to the lack of H_2_S, or the lack of one of its metabolites such as thiosulfate, or the accumulation of the 3-MST substrate 3-mercaptopyruvate remains to be investigated. Our results suggest that augmenting H_2_S production by 3-MST activation may be an approach to treat calcifying disorders.

## Data Availability

Data are available upon request to authors.

## Abbreviation

3-MST: 3-Mercaptosulfur transferase
CBS: Cystathionine beta-synthase
CSE: Cystathionine gamma-lyase
H_2_S: Hydrogen sulfide
NO: Nitric oxide
CO: Carbon monoxide
BCP: Basic calcium phosphate
HA: Hydroxyapatite
CPPD: Calcium pyrophosphate dihydrate
CPP: Calciprotein particles
MNX: Meniscectomy
ROS: Reactive oxygen species
NAC: N-acetyl cysteine
VOI: Volume of interests
OARSI: Osteoarthritis research society international
K/L: Kellgren-Lawrence
LDH: Lactate dehydrogenase
Alpl: Alkaline phosphatase
Ank: Progressive ankylosis protein homolog
Anx5: Annexin 5
Pit1: Phosphate transporter 1
Pit2: Phosphate transporter 2
MCDU: Beta-mercaptolactate cysteine disulfiduria
VSMCs: Vascular smooth muscle cells
IL-6: Interleukin-6
